# Alopecia Areata Associated with Dupilumab: National Database Study

**DOI:** 10.3390/diagnostics15141828

**Published:** 2025-07-21

**Authors:** Tarun Sontam, Humaira Nfn, Jodi Yanking Li, Sehar Nadeem, Katie Beier, Neil K. Jairath, Vignesh Ramachandran

**Affiliations:** 1 School of Medicine, Texas A&M College of Medicine, Bryan, TX 77807, USA; tarumsontram@tamu.edu; 2 Department of Biology, Macaulay Honors College at Brooklyn College, Brooklyn, NY 10023, USA; humairanfn@gmail.com; 3 Department of Psychology, New York University, New York, NY 10012, USA; jodiykli@gmail.com; 4 Department of Psychology, Brooklyn College, Brooklyn, NY 10023, USA; saiyrrn@outlook.com; 5 School of Medicine, Toledo Medical School, Toledo, OH 43614, USA; katie.beier@rockets.utoledo.edu; 6 Ronald O. Perelman Department of Dermatology, New York University, New York, NY 10016, USA; jairathn1996@gmail.com; 7 Skin Institute of New York, New York, NY 11375, USA

**Keywords:** alopecia areata, dupilumab, atopic dermatitis, adverse effect, Th2

## Abstract

**Background:** Alopecia areata (AA), an autoimmune condition causing non-scarring hair loss, often coexists with atopic dermatitis (AD) due to shared T-helper cell type 2 (Th2)-mediated pathways. Dupilumab, a monoclonal antibody inhibiting IL-4 and IL-13 signaling, is a cornerstone treatment for AD but has conflicting reports regarding its impact on AA, with some suggesting therapeutic benefits and others indicating AA induction. **Methods:** This retrospective study, utilizing the TriNetX Research Network’s de-identified data from over 300 million patient records, investigates the association between dupilumab use and AA risk in AD patients. **Results:** After propensity score matching, 23,782 dupilumab users were compared with an equal number of controls. Results revealed a statistically significant increased AA risk in dupilumab users (odds ratio: 1.436, 95% CI: 1.066–1.935, *p* = 0.0167) after 16 weeks. Cases occurring within 16 weeks were excluded. **Conclusions:** Potential mechanisms include immune rebalancing, with Th2 suppression possibly upregulating Th1/Th17 pathways or unmasking latent AA in predisposed individuals. These findings challenge dupilumab’s potential as an AA treatment and highlight the need for vigilant monitoring, including routine scalp examinations and patient education. Future research should focus on mechanistic pathways, risk stratification, and comparative studies with other biologics to optimize personalized treatment strategies for AD and AA.

## 1. Introduction

Alopecia areata (AA) is a prevalent autoimmune condition characterized by non-scarring hair loss, typically presenting as discrete, smooth patches on the scalp or other hair-bearing areas. Epidemiologically, AA affects approximately 2% of the global population, with a lifetime incidence of about 1 in 50 individuals, occurring across all age groups but most commonly in young adults and children [1]. First-degree relatives of AA patients have a 6–8% risk of developing the condition, highlighting a hereditary component [2]. Additionally, AA frequently coexists with atopic dermatitis (AD), with 15–20% of AA patients having concurrent AD, potentially due to shared T-helper cell type 2 (Th2)-mediated immunological pathways [3]. Up to 38% of AA patients may also have a personal or family history of atopy, including AD, asthma, or allergic rhinitis, which may amplify disease severity [4]. The condition is driven by immune dysregulation, predominantly involving Th2-mediated inflammation, with interleukin (IL)-4 and IL-13 playing critical roles in its pathogenesis [5].

The psychosocial impact of AA is profound, often leading to significant emotional distress and reduced quality of life. The visible nature of hair loss can cause embarrassment, anxiety, and depression, particularly in younger patients or those with extensive hair loss, such as alopecia totalis or universalis [6]. Studies indicate that up to 60% of AA patients experience clinically significant psychological distress, with severe cases correlating with higher rates of anxiety and depression [7]. The unpredictable course of AA exacerbates these challenges, contributing to social withdrawal and diminished self-esteem [8]. For patients with concurrent AD, the combined burden of visible skin and hair conditions can intensify these psychosocial effects, further impacting social interactions and mental well-being.

Dupilumab, a monoclonal antibody targeting the IL-4 receptor alpha subunit to inhibit IL-4 and IL-13 signaling, has revolutionized the management of moderate-to-severe AD by effectively reducing Th2-mediated inflammation [9]. It is a cornerstone treatment in dermatology, widely used for AD and approved for other Th2-driven conditions, such as chronic rhinosinusitis with nasal polyps, eosinophilic esophagitis, chronic spontaneous urticaria, and prurigo nodularis, with emerging indications for conditions like bullous pemphigoid [10]. Given the shared Th2-driven mechanisms between AA and AD, dupilumab has been hypothesized to benefit AA, with case reports documenting hair regrowth in some AA patients, particularly those with concurrent AD [11,12,13]. However, conflicting evidence from case reports and series suggests that dupilumab may paradoxically trigger AA in some cases [14,15]. This discrepancy raises critical questions about its role in AA, either as a therapeutic agent or a potential trigger.

This study herein leverages the power of a robust national real-world use database to provide a retrospective analysis to unravel the complex relationship between dupilumab treatment and the risk of alopecia areata (AA) diagnosis in patients with atopic dermatitis (AD). We discuss the results’ implications, postulate mechanisms of action, and encourage future directions for research.

## 2. Methods

The TriNetX Research Network (TriNetX, LLC, Cambridge, MA, USA) is a global platform that provides access to de-identified real-world data from over 300 million patient records across over 220 healthcare organizations in 30 countries, enabling advanced clinical and observational research [16]. For this study, we utilize TriNetX to retrospectively analyze the risk of alopecia areata in atopic dermatitis patients with and without dupilumab treatment, leveraging its robust dataset to inform personalized treatment strategies. From the TriNetX research network, all patients with 2 separate AD diagnoses (International Classification of Diseases-10 [ICD-10] L20.0) at least 30 days apart were eligible. Patients were then separated into 2 cohorts based on the presence of dupilumab prescription, with all patients prescribed any other Th2-targeting biologics (omalizumab, tralokinumab, mepolizumab, reslizumab, benralizumab, and rituximab) being excluded from analysis. Patients with a pre-existing diagnosis of AA (ICD-10 L63.0) were also excluded. The index date was the first prescription date of dupilumab for the treatment cohort or date of diagnosis of AD for the control cohort. Following exclusions, propensity score matching (PSM) was used to balance both cohorts on a variety of covariates (Table 1). The primary outcome assessed was the development of AA. As dupilumab can take up to 16 weeks to fully take effect, outcomes within 16 weeks of starting dupilumab were not considered. Statistical analysis was performed using IBM SPSS version 29 (Armonk, NY: IBM Corp.).

## 3. Results

After PSM, 23,782 dupilumab users were matched to an equal number of controls (Table 1). From at least 16 weeks from the index date, 105 dupilumab users developed AA while only 74 controls developed AA (odds ratio: 1.436, 95% confidence interval: 1.066, 1.935, *p*-value = 0.0167) (Table 1). This denotes statistical significance that dupilumab is associated with the development of alopecia areata in patients treated with this medication for AD.

## 4. Discussion

Our study, utilizing the TriNetX Research Network, unveils a statistically significant association between dupilumab use in atopic dermatitis (AD) patients and an increased risk of alopecia areata (AA), with an odds ratio of 1.436 (95% CI: 1.066–1.935, *p* = 0.0167) after propensity score matching (PSM) of 23,782 dupilumab users to an equal number of controls. This finding, derived from a large, well-matched cohort, represents a critical advancement in understanding dupilumab’s safety profile in real-world settings. The TriNetX platform, a global federated network aggregating de-identified data from over 300 million patient records across 220+ healthcare organizations in 30 countries, underpins the robustness of these results [17]. Its real-world data (RWD), refreshed by 80% of institutions every 1–4 weeks, include detailed prescription records, enabling precise tracking of dupilumab use and outcomes. The international scope ensures diverse patient demographics, enhancing generalizability, while standardized coding (e.g., ICD-10, RxNorm) and PSM for variables like age, sex, race, ethnicity, and comorbidities minimize confounding [16]. This study’s use of TriNetX’s longitudinal, high-fidelity data positions it as a landmark in real-world evidence (RWE), offering novel insights into the unintended consequences of biologic therapies in dermatology.

The clinical significance of our findings is amplified by ongoing clinical trials exploring dupilumab as a potential treatment for AA, given its role in modulating T-helper cell type 2 (Th2)-mediated inflammation, a shared pathway in AA and AD [18]. Trials such as NCT05551793 are evaluating dupilumab’s efficacy in AA, hypothesizing that its inhibition of interleukin (IL)-4 and IL-13 signaling could suppress autoimmune hair follicle damage. However, our results challenge this therapeutic optimism, suggesting that dupilumab may paradoxically precipitate AA in some AD patients. This finding has far-reaching implications for trial design and clinical practice. For instance, trials may need to incorporate risk stratification, excluding patients with a personal or family history of AA or other autoimmune conditions, in order to mitigate adverse events. Clinically, dermatologists must weigh dupilumab’s efficacy in AD against its potential to trigger AA, particularly in patients with atopic comorbidities, which affect up to 38% of AA cases [18]. The emergence of Janus kinase (JAK) inhibitors in both the AD and AA landscape offer alternatives for high-risk patients, potentially reshaping treatment algorithms [19,20,21]. Our study underscores the urgency of personalized medicine, urging clinicians to engage in nuanced risk–benefit discussions and prompting trialists to enhance safety monitoring protocols to capture AA as an adverse outcome.

The mechanisms by which dupilumab may trigger AA are complex and likely involve immune rebalancing within the skin’s microenvironment. AA is driven by a combination of Th2, Th1, and Th17 pathways, with IL-4 and IL-13 promoting Th2-mediated inflammation and interferon-gamma (IFN-γ) driving cytotoxic T cell infiltration in chronic cases [22]. Dupilumab’s blockade of IL-4 and IL-13 signaling via the IL-4 receptor alpha subunit should theoretically attenuate Th2-driven AA. However, our findings suggest a paradoxical effect, possibly due to compensatory upregulation of Th1 or Th17 pathways. For example, suppressing Th2 cytokines may enhance IFN-γ production, which recruits CD8+ T cells to hair follicles, initiating or exacerbating AA [22,23,24]. Alternatively, dupilumab may disrupt the cutaneous immune balance in AD patients with a genetic predisposition to autoimmunity, unmasking latent AA. Polymorphisms in IL-4R or Human Leukocyte Antigen (HLA) genes, implicated in both AD and AA, could modulate this risk, as seen in genome-wide association studies [24]. Another hypothesis posits that dupilumab alters hair follicle cycling, potentially inducing telogen effluvium that progresses to AA in susceptible individuals [25]. Systemic effects, such as changes in eosinophil levels or cytokine networks, may further contribute, as dupilumab has been linked to eosinophilia in some AD patients. There is the possibility that dupilumab may improve AA in Th2-dominant patients (e.g., those patients with high IgE or eosinophils) but may trigger AA in others by shifting immune balance toward Th1/Th17. These mechanisms highlight the need for translational research, including cytokine profiling and single-cell RNA sequencing, in order to pinpoint the molecular triggers of dupilumab-associated AA and identify biomarkers for at-risk patients.

Herein, in order to augment our results and provide a survey of the literature, we have performed a literature review for cases of AA after the initiation of dupilumab for AD. Our analysis of case reports in the literature are presented in Table 2. These results reveal critical trends that contextualize our findings and highlight areas for further exploration. First, most dupilumab-associated AA cases involve adults, with pediatric cases being notably underrepresented. This trend may reflect an age-related phenotypic susceptibility, possibly tied to differences in immune maturation or hair follicle biology, suggesting that adults may have a unique immunologic vulnerability to dupilumab-induced AA [26]. Alternatively, pediatric cases may be underdiagnosed due to less frequent scalp examinations or milder presentations, emphasizing the need for targeted screening in younger patients. Second, several case reports document AA onset before the 16-week threshold used in our study, which was deliberately set to align with dupilumab’s established efficacy endpoint in clinical trials and manufacturer guidelines, minimizing confounders like transient hair loss [27]. While early-onset cases suggest rapid immune dysregulation in susceptible individuals, our focus on post-16-week outcomes enhances specificity by capturing sustained, treatment-related AA. This dual perspective underscores the importance of monitoring across all treatment phases. Third, most case reports indicate hair regrowth following AA onset, often without discontinuing dupilumab, though a subset required treatment cessation for resolution. This variability highlights a pivotal clinical decision point: whether to continue dupilumab, anticipating spontaneous recovery, or to halt therapy to prevent persistent hair loss. These trends necessitate patient-centered discussions, balancing dupilumab’s benefits for AD control against the psychosocial and aesthetic impact of AA, and tailoring management based on individual response patterns. A framework to consider may be to trial conservative approaches to AA (topical corticosteroids, intralesional corticosteroids, +/− pulse oral corticosteroids); if there is no improvement after weekly or biweekly injections or there is rapid worsening, then it may be reasonable to consider holding dupilumab.

To address the risk of dupilumab-associated AA, we propose standardized clinical practices for screening and monitoring AD patients on dupilumab. Routine scalp examinations should be conducted at baseline, 8 weeks, 16 weeks, and every 3–6 months thereafter, using trichoscopy to detect early AA signs, such as yellow dots, black dots, or exclamation mark hairs [25]. Patient education is critical; we suggest clinicians inform patients about the potential risk of AA, encouraging self-monitoring and prompt reporting of hair loss. For high-risk patients—those with a personal or family history of AA, atopy, or autoimmune diseases—more frequent monitoring (e.g., monthly for the first 6 months) is recommended. Baseline scalp photography can serve as a reference for tracking changes, enhancing diagnostic precision. Integrating these practices into routine care ensures early detection, minimizing the psychosocial burden of AA, which affects up to 60% of patients with significant distress [7]. Dermatologists should also consider multidisciplinary collaboration with psychologists to address the emotional impact of hair loss, particularly in patients with concurrent AD and AA. These proactive measures balance dupilumab’s therapeutic benefits with vigilant monitoring, optimizing patient outcomes.

Future research is essential to confirm our findings and refine dupilumab’s risk–benefit profile. Prospective cohort studies should explore causality, incorporating longitudinal cytokine profiling and genomic sequencing to elucidate mechanistic pathways. Machine learning models, leveraging TriNetX’s vast dataset, could predict AA risk based on clinical, laboratory, demographic, and genetic variables, enabling precision risk stratification. In fact, a limiting factor in our study was the lack of laboratory data, including IgE levels and eosinophils counts, which may allow us to glean insights into which patients may be at risk for AA in the setting of dupilumab for AD. Subgroup analyses should investigate whether specific AD phenotypes (e.g., severe, head-and-neck predominant) or dupilumab dosing regimens influence AA risk. Comparative studies evaluating IL-13-specific biologics, such as tralokinumab, could clarify whether selective inhibition mitigates AA risk compared with a dual IL-4/IL-13 blockade. Qualitative research capturing patient experiences of dupilumab-associated AA could inform shared decision-making frameworks, enhancing patient-centered care. Additionally, international collaborations through TriNetX’s global network could facilitate rare-event analyses, particularly for pediatric cases, in order to address underreporting. By addressing these gaps, future research can empower clinicians to deliver tailored therapies, ensuring that dupilumab’s transformative potential in AD is harnessed safely.

## 5. Conclusions

In conclusion, our study, powered by the TriNetX Research Network, illuminates a novel risk of AA in AD patients treated with dupilumab, challenging its therapeutic promise in AA clinical trials. By proposing mechanistic insights, analyzing case report trends, and advocating for robust screening practices, we provide a roadmap for navigating this complex therapeutic landscape. As dermatology embraces precision medicine, our findings underscore the need to balance innovation with vigilance, ensuring that patients receive therapies optimized for their unique immunologic and clinical profiles. Continued research will be critical to identifying at-risk patients and refining treatment strategies, paving the way for safer, more effective management of AD and AA.

## Figures and Tables

**Table 1:** Baseline characteristics of dupilumab cohort and dupilumab-naive (control) cohort before and after propensity score matching (PSM). Bold standard difference (std diff) indicates std diff > 0.1.

	Baseline Characteristics Before PSM	Baseline Characteristics After PSM				
	Dupilumab Cohort (*n* = 23,820)	Control Cohort (*n* = 494,930)	Std Diff	Dupilumab Cohort (*n* = 23,782)	Control Cohort (*n* = 23,782)	Std Diff
Age at Index	31.7 ± 24.3	15.6 ± 21.7	**0.6988**	31.6 ± 24.2	33.1 ± 25.2	0.0616
Male	11,082 (46.5%)	238,661 (48.2%)	0.0340	11,061 (46.5%)	10,744 (45.2%)	0.0268
Female	12,306 (51.7%)	247,229 (50.0%)	0.0342	12,290 (51.7%)	12,630 (53.1%)	0.0286
White	11,061 (46.4%)	180,134 (36.4%)	**0.2049**	11,033 (46.4%)	11,166 (47.0%)	0.0112
Black or African American	4965 (20.8%)	124,583 (25.2%)	**0.1030**	4961 (20.9%)	4751 (20.0%)	0.0219
Nicotine Dependence	1666 (7.0%)	12,006 (2.4%)	**0.2169**	1662 (7.0%)	1614 (6.8%)	0.0080
Systemic Lupus Erythematosus	155 (0.7%)	1598 (0.3%)	0.0471	155 (0.7%)	120 (0.5%)	0.0194
Psoriasis	1426 (6.0%)	7307 (1.5%)	**0.2397**	1412 (5.9%)	1364 (5.7%)	0.0086
Asthma	7089 (29.8%)	91,270 (18.4%)	**0.2670**	7075 (29.7%)	6944 (29.2%)	0.0121
Allergic Rhinitis	7451 (31.28%)	112,508 (22.7%)	**0.1934**	7443 (31.3%)	7469 (31.4%)	0.0024
Type 1 Diabetes Mellitus	239 (1.0%)	2709 (0.5%)	0.0520	239 (1.0%)	158 (0.7%)	0.0374
Food Allergies	4629 (19.4%)	49,425 (10.0%)	**0.2691**	4621 (19.4%)	4515 (19.0%)	0.0113
Autoimmune Thyroiditis	192 (0.8%)	1829 (0.4%)	0.0571	192 (0.8%)	147 (0.6%)	0.0225
Topical Glucocorticoid Use	20,724 (87.0%)	345,720 (69.9%)	**0.4263**	20,686 (87.0%)	20,591 (86.6%)	0.0118
Methotrexate Use	2042 (8.6%)	3675 (0.7%)	**0.3782**	2004 (8.4%)	1705 (7.2%)	0.0469
# of Eosinophils/100 Leukocytes in Blood	6859 (28.8%)	41,615 (8.4%)	**0.5429**	6824 (28.7%)	6764 (28.4%)	0.0056

**Table 2:** Literature review and summary of cases illustrating alopecia areata as an adverse event in the setting of dupilumab use.

Case (Year)	Age/Sex	Site	Dupilumab Indication	Comorbidities	Onset	Treatment	Management/Outcome
Flanagan et al. (2019) [15]	27 years old/male	AA on scalp (vertex and temporal region)	Moderate AD	N/A	18 weeks	Loading dose of 600 mg, dupilumab, 300 mg subcutaneously AA—intralesional triamcinolone and clobetasol	Complete hair regrowth 2 months after dupilumab discontinued
Gallo (2020) [28]	24 years old/male	Entire scalp	Severe AD	Ulcerative colitis	8 weeks	Loading dose of 600 mg, dupilumab, 300 mg subcutaneously AA—dupilumab was discontinued; topical clobetasol cyclosporine 3 mg/kg/day	Partial hair regrowth within 4 weeks; after 3 months, hair regrowth was complete
Kulkarni et al. (2022) [29]	22 years old/male	Patchy AA on vertex scalp	Moderate AD	N/A	3–4 months	Initially, 300 mg Q2W, later switched to 300 mg Q4W AA—oral/topical steroids + mycophenolate	Complete regrowth 5–6 months after dupilumab discontinued; no relapse of AA after dupilumab reintroduced with lower dose
Mitchell & Levitt (2018) [14]	29 years old/male	Patchy AA on scalp	Chronic AD	N/A	5 weeks	300 mg Q2W AA—intralesional triamcinolone (10 mg/mL)	Partial hair regrowth
Carnicle et al. (2021) [30]	42 years old/female	Reactivation of AA (diffuse, androgenetic-like pattern)	Severe AD	AA in remission for >5 years	4 months	Not specified AA—1 dose IM triamcinolone	Complete hair regrowth 2 months after dupilumab discontinued
Chromy et al. (2023) [31]	36 years old/male	AA of beard	Chronic rhinosinusitis with nasal polyps (No AD)	N/A	25 weeks	Not specified	N/A
Barroso-Garcia et al. (2018) [32]	31 years old/male	AA in patches on anterior scalp	Severe AD	N/A	6 weeks	Initially, 600 mg followed by 300 mg every 2 weeks AA—intralesional triamcinolone	N/A
Salgüero-Fernández et al. (2018) [33]	33 years old/male	Diffuse AA in frontal and occipital region + beard	Severe AD	N/A	7 weeks	Initially, 600 mg followed by 300 mg every 2 weeks AA—topical mometasone 0.1%	Complete hair regrowth after 3 months
Yazdanyar et al. (2019) [34]	24 years old/male	AA in patches	AD (unspecified severity)	N/A	1 week	Initially, 600 mg followed by 300 mg every 2 weeks AA—ketoconazole 3% shampoo	Partial hair regrowth after 3 weeks
Barbarin et al. (2019) [35]	23 years old/female	AA in patches in frontal, vertex, and occipital areas	Chronic AD	N/A	48 h	Initially, single 600 mg dose, stopped after 8 weeks of treatment AA—topical minoxidil and clobetasol propionate	Complete hair regrowth after 6 months
Kanda et al. (2019) [36]	35 years old/male	AA in patches, mostly in parietal, occipital, and frontal regions	Severe AD	N/A	6 weeks	Initially, 600 mg followed by 300 mg every 2 weeks AA—methylprednisolone 0.5 g/day for 3 days	Partial hair regrowth (78%) after 4 months
Stander et al. (2020) [37]	53 years old/male	AA in patches	Recurrent AD	N/A	1 year	Initially, 600 mg followed by 300 mg every 2 weeks AA—dupilumab was discontinued; cyclosporine 200 mg/day	Complete hair regrowth after 4 months
Beaziz et al. (2021) [38]	45 years old/female	AA in oval patches in occipital region + two small patches in temporal region	Severe AD	Asthma and allergic rhinitis	1 year	Initially, 600 mg followed by 300 mg every 2 weeks AA—clobetasol propionate 0.05%	Complete hair regrowth after 2 months
Chung et al. (2019) [39]	51 years old/female	AA in patches	Chronic AD	At 26 months, patient noted generalized thinning of hair; suspected for iron deficiency-induced telogen effluvium (no improvement after successful iron supplementation)	28 months (AA patches first appeared)	24 months of open-label dosing in clinical trials of dupilumab; then transitioned to commercial dosing of dupilumab 300 mg every 2 weeks	At 30 months, hair loss progressed to AU (alopecia universalis) At 34 months, patient resumed dupilumab monthly shot At 44 months, 90% regrowth of scalp hair
Chung et al. (2019) [39]	25 years old/male	Alopecia universalis, loss of his few remaining patches of scalp hair	Chronic AD	N/A	6–8 weeks	AA—dupilumab was discontinued; cyclosporine and tofacitinib	Patchy improvement of alopecia totalis within 6 weeks of treatment
Zhu (2020) [40]	37 years old/male	Right jawline, localized non-scarring alopecic patch	AD (unspecified severity)	N/A	5 weeks	Dupilumab 300 mg Q2W AA—topical calcineurin inhibitors	No follow-up visit
Zhu (2020) [40]	37 years old/female	Scalp, occipital localized alopecic patch	AD (unspecified severity)	Pre-existing alopecia areata	N/A (Prior resolved alopecia; recurrent on dupilumab)	300 mg Q2W AA–topical steroids	Continued dupilumab; stable disease

## Data Availability

Data available upon request from the corresponding author.

## Abbreviations

AA	alopecia areata
AD	atopic dermatitis
IL	interleukin
HLA	human leukocyte antigen
ICD	International Classification of Diseases
